# A Decade of Antimicrobial Resistance in Human and Animal *Campylobacter* spp. Isolates

**DOI:** 10.3390/antibiotics13090904

**Published:** 2024-09-21

**Authors:** Rita Barata, Maria José Saavedra, Gonçalo Almeida

**Affiliations:** 1 National Institute of Agricultural and Veterinary Research (INIAV), 4485-655 Vila do Conde, Portugal; anarita.barata@iniav.pt; 2 Centre for the Research and Technology of Agro-Environmental and Biological Sciences (CITAB), Institute for Innovation, Capacity Building and Sustainability of Agri-Food Production (Inov4Agro), University of Trás-os-Montes and Alto Douro, 5001-801 Vila Real, Portugal; saavedra@utad.pt; 3 Center for Veterinary and Animal Research (CECAV), Associated Laboratory of Animal and Veterinary Science (AL4AnimalS), University of Trás-os-Montes and Alto Douro, 5000-801 Vila Real, Portugal; 4 AB2Unit—Antimicrobials, Biocides & Biofilms Unit, Veterinary Sciences Department, University of Trás-os-Montes and Alto Douro (UTAD), 5001-801 Vila Real, Portugal; 5 Center for Animal Science Studies (CECA-ICETA), Associated Laboratory of Animal and Veterinary Science (AL4AnimalS), University of Porto, 4099-002 Porto, Portugal

**Keywords:** *Campylobacter*, one health, resistance patterns, campylobacteriosis, antimicrobial global threats

## Abstract

**Objectives**: *Campylobacter* spp. remain a leading cause of bacterial gastroenteritis worldwide, with resistance to antibiotics posing significant challenges to treatment and public health. This study examines profiles in antimicrobial resistance (AMR) for *Campylobacter* isolates from human and animal sources over the past decade. **Methods**: We conducted a comprehensive review of resistance data from studies spanning ten years, analyzing profiles in resistance to key antibiotics, ciprofloxacin (CIP), tetracycline (TET), erythromycin (ERY), chloramphenicol (CHL), and gentamicin (GEN). Data were collated from various regions to assess global and regional patterns of resistance. **Results**: The analysis reveals a concerning trend of increasing resistance patterns, particularly to CIP and TET, across multiple regions. While resistance to CHL and GEN remains relatively low, the high prevalence of CIP resistance has significantly compromised treatment options for campylobacteriosis. Discrepancies in resistance patterns were observed between human and animal isolates, with variations across different continents and countries. Notably, resistance to ERY and CHL showed regional variability, reflecting potential differences in antimicrobial usage and management practices. **Conclusions**: The findings underscore the ongoing challenge of AMR in *Campylobacter*, highlighting the need for continued surveillance and research. The rising resistance prevalence, coupled with discrepancies in resistance patterns between human and animal isolates, emphasize the importance of a One Health approach to address AMR. Enhanced monitoring, novel treatment strategies, and global cooperation are crucial for mitigating the impact of resistance and ensuring the effective management of *Campylobacter*-related infections.

## 1. Introduction

*Campylobacter* is a Gram-negative bacterium that is associated with gastroenteritis and enterocolitis in humans worldwide [1]. In the European Union (EU), campylobacteriosis has been the most reported gastrointestinal infection in humans since 2005 [2].

The species most associated with human disease are *C. jejuni* and *C. coli*, both recognized for causing gastrointestinal infections, particularly gastroenteritis [3]. Symptoms typically emerge 2 to 5 days after bacterial ingestion and can persist for up to a week, predominantly presenting as gastroenteritis. However, the infection may progress to more serious complications, such as Guillain–Barré syndrome, an autoimmune condition leading to paralysis, as well as reactive arthritis, bacteremia, and, in rare instances, endocarditis [4]. The pathogenicity of *Campylobacter* is linked to its ability to invade the intestinal epithelium, produce toxins, and elicit a robust inflammatory response. This can heighten the risk of severe complications, depending on the infection’s intensity and the host’s overall health condition [5].

Recent studies on the sources of infection suggest that, in addition to poultry, ruminants also play a significant role in human campylobacteriosis [6]. *C. jejuni* accounts for over 95% of clinical cases, with undercooked meat being the primary source of contamination [7]. The presence of these bacteria throughout the meat production chain poses a public health risk, creating significant challenges for health authorities in terms of monitoring, underreporting, and control [8].

Although antibiotic treatment is generally not required for gastroenteritis, as campylobacteriosis often resolves without intervention, it can reduce the duration of illness when the antimicrobial drugs are effective against the specific *Campylobacter* strains involved in the infection [9]. However, in cases where symptoms persist or when patients are at higher risk of severe complications, antibiotic therapy is recommended [10]. Macrolides, such as erythromycin (ERY), and fluoroquinolones like ciprofloxacin (CIP), are the first and second-line choices of treatment, respectively. When campylobacteriosis is caused by antibiotic-resistant *Campylobacter* strains, suboptimal treatment outcomes or even failure may occur. In such situations, alternative antibiotics such as tetracycline (TET) and gentamicin (GEN), or amphenicols such as chloramphenicol (CHL) may be used [11]. Notably, fluoroquinolone-resistant *Campylobacter* has been included on the WHO’s list of high-priority pathogens for research and the development of new antibiotics [12].

Antimicrobial resistance (AMR) is defined by Prestinaci et al. [13] as the reduced or complete inability of an antimicrobial agent to inhibit bacterial growth, which, in the case of pathogenic organisms, may result in treatment failure. AMR in food-producing animals can be transmitted to humans through foodborne pathways, as observed with bacteria such as *Campylobacter* spp., *Salmonella* spp., and *Escherichia coli*, as well as through environmental contamination and direct contact with animals [14]. Several factors contribute to the spread of resistance, including the improper use of antimicrobial agents in both human and veterinary medicine, and inadequate hygiene practices in healthcare settings and throughout the food production chain, which facilitate the transmission of resistant strains. This progression reduces the efficacy of antimicrobials, potentially rendering them ineffective over time [15].

This study aimed to compare the antimicrobial resistance of *Campylobacter* isolates from human and animal sources against key antibiotics, including CHL, CIP, ERY, GEN, and TET. The objectives were to understand the dynamics of resistance frequency, explore the factors that contribute to its increase or decrease, and investigate whether antimicrobial AMR in food-producing animals could be transmitted to humans through food.

## 2. Results

### 2.1. Studies of Antimicrobial Resistance of Campylobacter spp.

In total, 147 research articles met the inclusion criteria, each describing the prevalence of antimicrobial-resistant *Campylobacter* spp. isolates derived from humans and/or animal samples collected from food-producing animals, including broilers, pigs, and ruminants. Of the 147 studies deemed eligible for further analysis, 28 exclusively reported on human isolates, 103 focused solely on animal isolates, and 16 covered both types.

The articles that discussed human isolates typically involved stool samples from both symptomatic and asymptomatic patients with campylobacteriosis. Regarding the animal isolates, 98 studies involved samples from broilers, including those taken from the ceca, carcasses, and meat. In the 23 studies concerning pig isolates, samples of feces and meat were analyzed. Similarly, the 28 studies focusing on ruminant isolates also used feces and meat samples.

Of the 147 studies analyzed, 49 employed the disk diffusion methodology (DD), 97 used the minimal inhibitory concentration methodology (MIC), and one study utilized both methodologies.

#### 2.1.1. Studies on Antibiotic Resistance in Human Isolates of *Campylobacter* spp.

**Figure 1:**
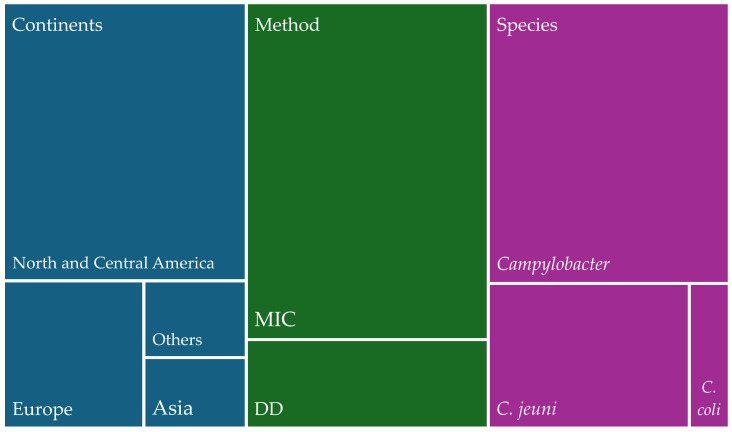
Distribution of studies on human isolates of *Campylobacter* spp. by world region, methodologies used, and species identified.

A total of 44 articles were used to gather information on human isolates of resistant *Campylobacter* spp. (Figure 1). Regarding the geographical origin of the isolates, 4 studies were from Africa, 8 were from Asia, 21 were from Europe, 6 were from North and Central America, 3 were from South America, and 2 were from Oceania. Of the two methodologies used, the one most used for human isolates was MIC, which was described in 30 articles; DD was described in 13, and one study used both. Of the articles that investigated the species of the isolates found, *C. jejuni* was the most reported species. Regarding isolates considered as multidrug-resistant (MDR), of the 19,908 human isolates, only a Spanish study conducted in 2021 revealed that among 26 isolates, only 1 (3.9%) was confirmed as MDR.

#### 2.1.2. Studies on Antibiotic Resistance in Animal Isolates of *Campylobacter* spp.

**Figure 2:**
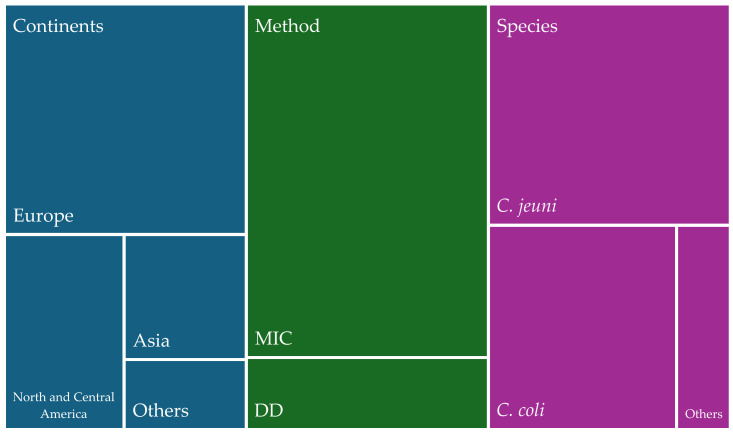
Distribution of studies on animal isolates (%) of *Campylobacter* spp. by world region, methodologies used, and species identified.

Among the 119 articles providing information on animal isolates of antibiotic-resistant *Campylobacter* isolates (Figure 2), the distribution across continents was as follows: 13 articles pertained to Africa, 25 to Asia, 61 to Europe, 11 to North and Central America, 8 to South America, and 1 to Oceania. Regarding the methodologies employed, the MIC technique was the most used, accounting for 65.6% of the studies. As for the species of *Campylobacter* spp. isolated, *C. jejuni* was the most frequently reported in animal samples. Taking into account all the animal isolates from the studies, we calculated that 15.7% of the animal isolates were MDR.

**Figure 3:**
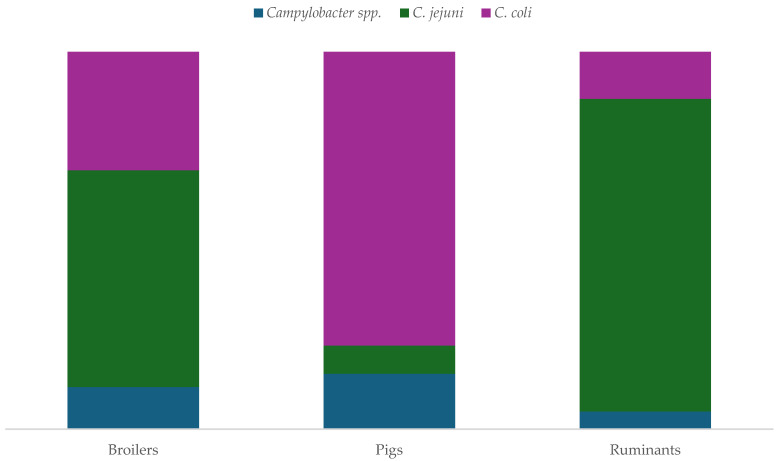
Distribution of animal isolates (%) of *Campylobacter* spp. by animal type and species identified.

When examining the distribution data by type of animal (broilers, pigs, and ruminants), notable differences emerged regarding the prevalence of *Campylobacter* spp. isolates, as shown in Figure 3. Concerning MDR, in chicken samples, *C. jejuni* was the most prevalent species, with 22% of the isolates being MDR. In contrast, in swine samples, *C. coli* emerged as the most prevalent species with 22% of the isolates being MDR. Lastly, in ruminant samples, *C. jejuni* was the most prevalent species, with 7% of the usolates being MDR.

### 2.2. Resistance of Campylobacter spp. Isolates from African Studies

Studies conducted in Benin, Ethiopia, Côte d’Ivoire, Kenya, Morocco, South Africa, Tanzania, and Tunisia are summarized in Table 1.

In Benin, in 2022, TET showed the highest resistance among animal isolates, with over 90% classified as MDR.

In Ethiopia, between 2014 and 2021, there was an increase in resistance to CIP, ERY, GEN, and TET among human isolates, with TET showing the highest resistance. Resistance to CHL decreased. For animal isolates, from 2021 to 2022, resistance to CIP and TET decreased, while resistance to CHL increased. By 2022, over 84% of animal isolates were classified as MDR.

In Côte d’Ivoire, a study of 76 animal isolates revealed high resistance to CIP.

In Kenya, data from 2021 showed that ERY had the highest resistance among 18 human isolates, while CIP was the most resistant among 35 animal isolates. In 2022, over 54% of animal isolates were classified as MDR.

In Morocco, a study of 143 animal isolates identified TET with the highest resistance level.

In South Africa, an analysis of 464 animal isolates between 2020 and 2021 showed increased resistance to all five antibiotics studied, with ERY showing the highest resistance. By 2022, over 87% of animal-origin isolates were classified as MDR.

In Tanzania, data from 2015 showed increased resistance to CIP and ERY among 136 human isolates. Between 2015 and 2016, resistance also increased among 134 animal isolates, with over 47% classified as MDR in 2016.

Finally, in Tunisia, a comparison of 180 animal isolates from 2018 to 2022 showed decreased resistance to CIP and ERY, while resistance to TET remained at 100%. The percentage of MDR isolates decreased from 100% to 37.5% by 2022.

**Figure 4:**
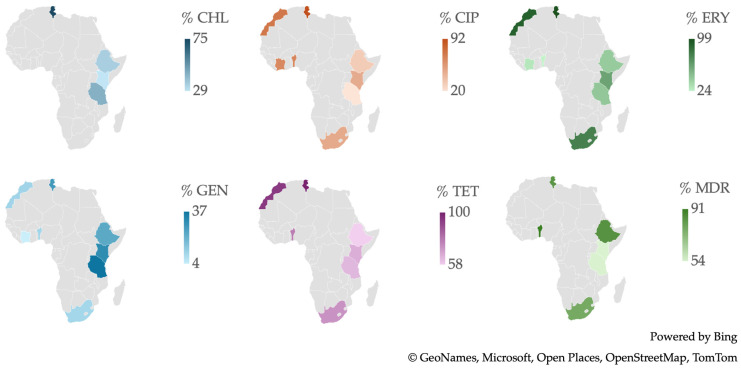
Antibiotic resistance patterns of *Campylobacter* spp. isolates from human and animal data from Africa.

From a geographical standpoint (Figure 4), Tunisia is distinguished by having the highest number of isolates resistant to a broad spectrum of antibiotics, including CHL, CIP, ERY, and TET. Tanzania recorded the highest percentage of resistance to GEN, while Benin is particularly noteworthy for having the highest percentage of MDR isolates.

**Figure 5:**
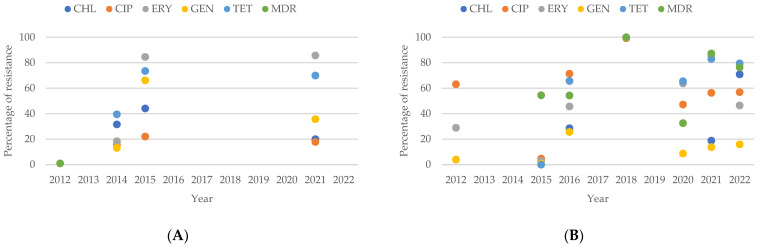
(**A**) Antibiotic resistance patterns of *Campylobacter* spp. Isolates from humans from Africa between 2012 and 2022. (**B**) Antibiotic resistance patterns of *Campylobacter* spp. Isolates from animals from Africa between 2012 and 2022.

The resistance profiles of *Campylobacter* spp. from human sources to CHL, CIP, ERY, GEN, and TET from 2012 to 2022 are shown in Figure 5A. ERY and TET have consistently been the antibiotics to which the highest number of isolates are resistant over the longest period. Resistance to CHL, CIP, and GEN decreased between 2015 and 2021, although there was a notable increase in resistance to all studied antibiotics between 2014 and 2015.

Figure 5B presents the resistance patterns of *Campylobacter* spp. from animal sources for the same antibiotics over the same period. The most recent data from 2022 show the highest levels of resistance to CHL since 2012. Additionally, there is a noticeable difference in resistance percentages between TET and CIP compared to GEN.

### 2.3. Resistance of Campylobacter spp. Isolates from Asian Studies

Data on *Campylobacter* spp. resistance from studies conducted in China, India, Japan, Jordan, Korea, Pakistan, the Philippines, and Thailand are summarized in Table 2.

In China, resistance to TET increased among human isolates from 2014 to 2022, while resistance to other antibiotics declined. Animal isolates showed rising resistance to CHL, CIP, and TET, with over 90% classified as MDR in 2021.

In India, from 2013 to 2021, human isolates exhibited the highest resistance to CIP, while animal isolates showed increasing resistance to CIP, ERY, GEN, and TET, with 41.5% classified as MDR in 2021.

In Japan, human isolates in 2019 had the highest resistance to CIP. Animal isolates from 2012 to 2017 showed decreasing resistance to CHL and ERY, with TET remaining the most resistant antibiotic.

In Jordan (2012), human isolates exhibited high resistance to CIP, ERY, and TET.

In Korea, TET had the highest resistance among human isolates in 2013, while animal isolates from 2017 to 2021 showed increasing resistance to all five antibiotics studied, with over 75% classified as MDR.

In Pakistan, TET was the antibiotic with the highest resistance among human isolates in 2018.

In the Philippines, animal isolates from 2014 to 2017 showed increasing resistance to ERY, GEN, and TET, with over 71% classified as MDR by 2017.

In Thailand, resistance among animal isolates to CIP, ERY, GEN, and TET increased from 2013 to 2021, with CIP showing the highest resistance by 2021.

**Figure 6:**
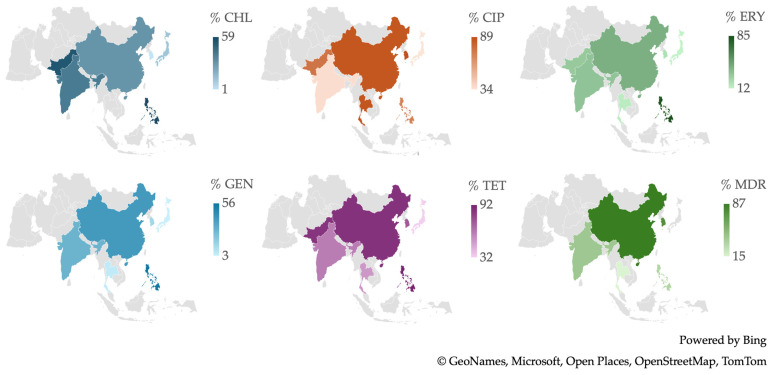
Antibiotic resistance patterns of *Campylobacter* spp. isolates from human and animal data from Asia.

From a geographical perspective, as illustrated in Figure 6, the Philippines is notable for having the highest number of *Campylobacter* spp. isolates resistant to a wide range of antibiotics, including CHL, ERY, GEN, and TET. South Korea recorded the highest percentage of resistance to CIP, while China stands out for having the highest percentage of MDR isolates.

**Figure 7:**
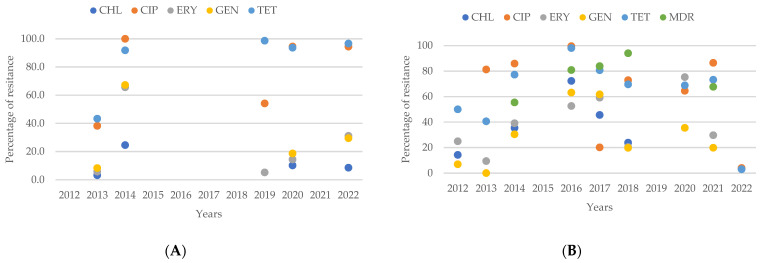
(**A**) Antibiotic resistance patterns of *Campylobacter* spp. isolates from humans from Asia between 2012 and 2022. (**B**) Antibiotic resistance patterns of *Campylobacter* spp. isolates from animals from Asia between 2012 and 2022.

The analysis of resistance patterns in *Campylobacter* spp. from human sources, as shown in Figure 7A, reveals that from 2012 to 2022, the number of isolates resistant to CHL, ERY, and GEN consistently remained lower than those resistant to CIP and TET. In contrast, the resistance patterns for *Campylobacter* spp. from animal sources, presented in Figure 7B, show increasing percentages of resistance to CIP, ERY, GEN, and TET from 2012 to 2014. When comparing resistance patterns between human and animal isolates over this period, it is evident that animal isolates exhibit a higher percentage of resistance to CHL, ERY, and GEN compared to human isolates.

### 2.4. Resistance of Campylobacter spp. Isolates from European Studies

Data on *Campylobacter* spp. isolates from studies across Europe, including Austria, Belgium, Croatia, Czechia, Estonia, Finland, France, Germany, Greece, Ireland, Italy, Latvia, Lithuania, Romania, North Macedonia, Poland, Portugal, Spain, Sweden, and the UK, are presented in Table 3.

In most countries, CIP resistance was predominant among both human and animal isolates. For instance, in Austria (2016) and Czechia (2018), CIP had the highest resistance in both human and animal isolates, with some studies reporting a high frequency of MDR, such as Czechia where 60% of animal isolates were MDR.

In Belgium, between 2017 and 2020, human isolates showed increasing resistance to CIP, ERY, and TET, while resistance among animal isolates decreased for these antibiotics, except for GEN, which saw an increase. In Croatia and Estonia, CIP also exhibited the highest resistance levels for human isolates.

Finland, France, Germany, and Greece similarly found CIP as the leading antibiotic for resistance, although France reported a decrease in CIP resistance in animal isolates between 2015 and 2017. Notably, Germany and Greece showed increasing patterns in resistance to CIP, GEN, and TET.

In Ireland (2022), Italy, and Lithuania (2022), resistance to TET was highest among animal isolates, with significant MDR percentages reported. Poland, Portugal, and Spain all reported increasing resistance to CIP, ERY, and TET in both human and animal isolates, with Spain showing a marked increase in MDR animal isolates.

In Romania and Sweden, resistance to CIP decreased over time, while resistance to TET increased. Similarly, UK data showed TET as the antibiotic with the highest resistance among animal isolates.

**Figure 8:**
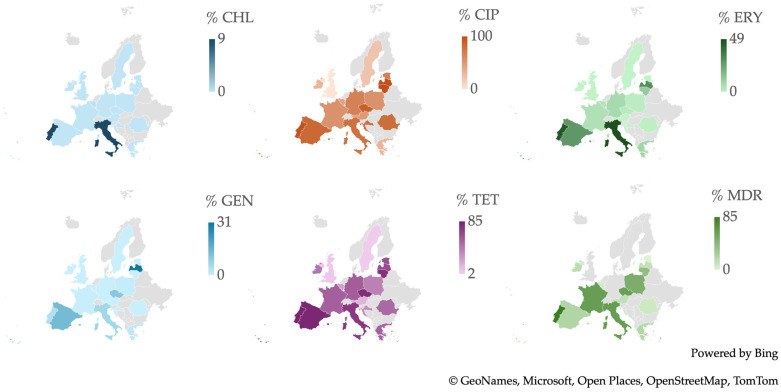
Antibiotic resistance patterns of *Campylobacter* spp. isolates from human and animal data from Europe.

From a geographic perspective, as illustrated in Figure 8, Italy, Latvia, and Portugal stand out prominently. Italy and Portugal reported the highest percentage of resistance to CHL, with a frequency of resistance reaching 9.2%. Latvia and Lithuania exhibited a striking 100% frequency of resistance to CIP. Italy also had the highest percentage of resistance to ERY at 49.0%, while Latvia reported the highest resistance to GEN at 31.1%. Additionally, Portugal demonstrated the highest percentage of resistance to TET at 85.2% and had the highest percentage of MDR isolates at 85.1%.

When analyzing the percentages of *Campylobacter* spp. isolates resistant to CHL and ERY across European countries, a higher frequency of resistance was observed in Southern European nations, particularly in the Mediterranean region, which includes Portugal, Spain, Italy, and Greece. In contrast, Northern European countries, such as Estonia, Latvia, Lithuania, Denmark, Finland, Iceland, Norway, and Sweden, exhibited lower resistance percentages. A similar trend was noted for resistance to CIP and TET, though it was less pronounced due to broader resistance across more European countries toward these antibiotics, which were the common resistance phenotypes among *Campylobacter* spp. isolates. GEN showed the lowest frequency of resistance among European isolates, with the highest resistance observed in Latvia. Additionally, Southern European countries had the highest percentage of isolates classified as MDR.

Comparing these findings with the EFSA report [15], Greece and Spain were found to have the highest frequency of resistance contamination for *Campylobacter* spp. in food, at 100% and 65.3%, respectively.

**Figure 9:**
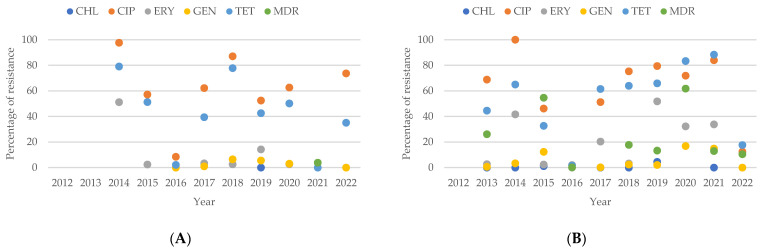
(**A**) Antibiotic resistance patterns of *Campylobacter* spp. isolates from humans from Europe between 2012 and 2022. (**B**) Antibiotic resistance patterns of *Campylobacter* spp. isolates from animals from Europe between 2012 and 2022.

By analyzing the resistance prevalence of *Campylobacter* spp. from human sources, as shown in Figure 9A, for the antibiotics CHL, CIP, ERY, GEN, and TET from 2012 to 2022, a distinct difference is observed between the resistance percentages for CIP and TET compared to CHL, ERY, and GEN, with CIP and TET showing higher resistance levels. Notably, from 2014 to 2022, resistance to CIP consistently exceeded that of TET.

In contrast, the analysis of resistance patterns for *Campylobacter* spp. from animal sources, depicted in Figure 9B over the same period, shows that the difference between resistance percentages for CIP and TET compared to CHL, ERY, and GEN is less pronounced, with CHL and GEN resistance remaining the lowest. Unlike in human isolates, the resistance percentage to CIP in animal isolates does not consistently exceed that of TET.

When comparing resistance percentages by year, animal isolates showed lower resistance to TET and ERY compared to human isolates in 2014. Similarly, in 2015, animal isolates had lower resistance percentages for TET and CIP. By 2017, resistance to TET was higher in animal isolates, while resistance to CIP was lower. From 2018 to 2021, animal isolates exhibited higher resistance percentages for both TET and CIP compared to human isolates. However, in 2022, these percentages were reversed, with animal isolates showing lower resistance to TET and CIP compared to human isolates. Additionally, the percentage of isolates resistant to ERY has consistently been higher in animal isolates.

### 2.5. Resistance of Campylobacter spp. Isolates from Northern and Central American Studies

Data on *Campylobacter* spp. resistance from North and Central America, including Canada, Grenada, Guatemala, Mexico, and the United States (US), are summarized in Table 4.

In Canada, between 2012 and 2019, resistance patterns varied between human and animal isolates. Among animal isolates, resistance to CIP, ERY, and GEN increased, while TET showed the highest resistance by 2019. Human isolates displayed increasing resistance to TET, which peaked in 2015. MDR in animal isolates was reported at 4.3% in 2012.

In Grenada (2014) and Guatemala (2014), TET was the antibiotic with the highest resistance among animal and human isolates, respectively.

In Mexico (2012), CIP had the highest resistance in human isolates, while TET was the most resistant among animal isolates.

Finally, in the US, CIP was the antibiotic to which most human isolates were resistant in 2017. Between 2014 and 2022, resistance percentages for all tested antibiotics increased, with TET showing the highest resistance in animal isolates by 2022, and 16.7% classified as MDR.

**Figure 10:**
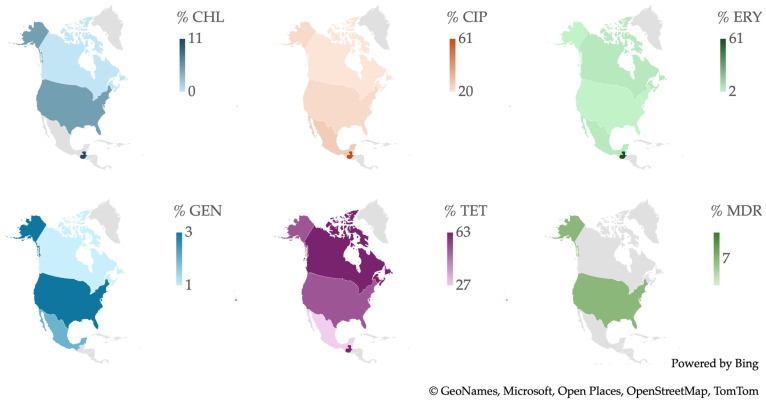
Antibiotic resistance patterns of *Campylobacter* spp. isolates from human and animal data from North and Central America.

From a geographic perspective, as shown in Figure 10, Guatemala stands out with the highest percentage of isolates resistant to CHL, CIP, and ERY. The USA has the highest percentage of isolates resistant to GEN and the highest percentage of MDR isolates. Finally, Canada has the highest percentage of isolates resistant to TET.

**Figure 11:**
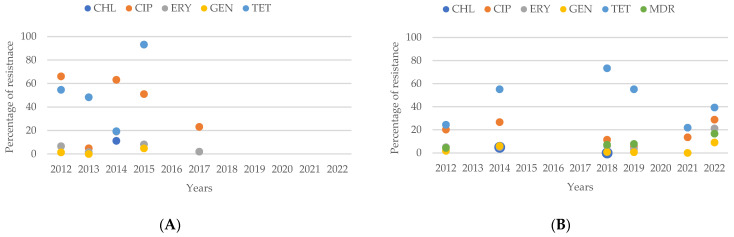
(**A**) Antibiotic resistance patterns of *Campylobacter* spp. isolates from humans from North and Central America between 2012 and 2022. (**B**) Antibiotic resistance patterns of *Campylobacter* spp. isolates from animals from North and Central America between 2012 and 2022.

Analyzing the resistance patterns of *Campylobacter* spp. from human sources, in Figure 11A for the antibiotics CHL, CIP, ERY, GEN, and TET from 2012 to 2022, we can see a clear difference between the percentages of resistance for CIP and TET compared to ERY and GEN, with CIP and TET showing higher resistance levels. From 2012 to 2017, resistance to CIP showed a decreasing trend.

For *Campylobacter* spp. from animal sources, the resistance prevalence for the same antibiotics over the same period in Figure 11B shows that TET was consistently the antibiotic to which the largest number of isolates showed resistance.

Comparing the percentages of resistant isolates from human and animal sources in 2012 and 2014, it is evident that in 2012, the percentage of human isolates resistant to CIP and TET was higher than that of animal isolates. However, in 2014, the percentage of animal isolates resistant to TET was higher compared to human isolates, while resistance to CIP remained lower.

### 2.6. Resistance of Campylobacter spp. Isolates from Southern American Studies

Data on *Campylobacter* spp. resistance in South America, covering Brazil, Chile, Ecuador, and Peru, are summarized in Table 5.

In Brazil (2013–2020), resistance percentages to all five antibiotics decreased among 176 animal isolates. In 2020, CIP had the highest resistance, with 35.7% of isolates classified as MDR.

In Chile, 354 isolates (7 human, 347 animal) were analyzed. Human isolates in 2016 showed the highest resistance to CIP and TET. Among animal isolates, resistance to CIP, GEN, and TET decreased between 2016 and 2017, while resistance to ERY increased. A 2016 comparison indicated higher resistance in human isolates for all antibiotics.

In Ecuador (2018), CIP was the antibiotic with the highest resistance among 218 animal isolates.

In Peru (2017–2019), 1032 animal isolates were examined, showing a decrease in resistance to CIP, ERY, and TET, while GEN resistance increased. CIP had the highest resistance in 2018.

**Figure 12:**
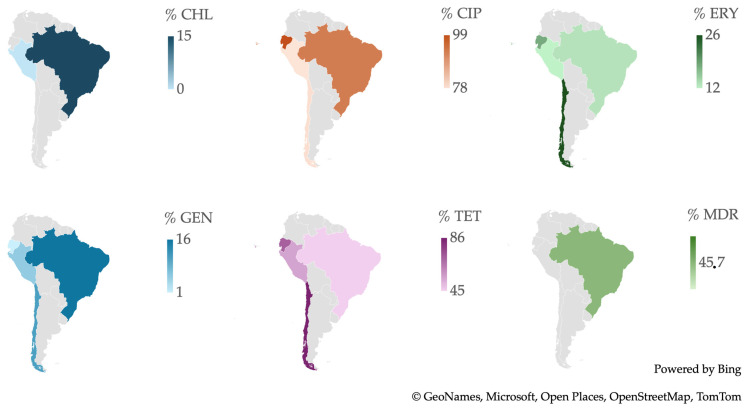
Antibiotic resistance patterns of *Campylobacter* spp. isolates from South America.

From a geographic perspective, as shown in Figure 12, Brazil in South America has the highest percentage of resistance to CHL and GEN and is the only country that reported isolates that are considered MDR, with a frequency of resistance of 45.7%. Chile has the highest percentage of resistance to ERY and TET. Ecuador has the highest percentage of resistance to CIP.

**Figure 13:**
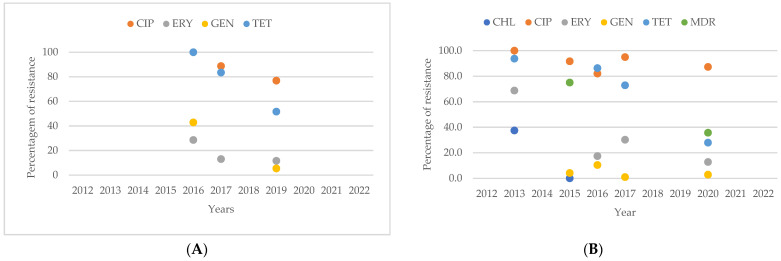
(**A**) Antibiotic resistance patterns of *Campylobacter* spp. isolates from humans from South America between 2012 and 2022. (**B**) Antibiotic resistance patterns of *Campylobacter* spp. isolates from animals from South America between 2012 and 2022.

Observing the prevalence of resistance in *Campylobacter* spp. isolates from human sources, in Figure 13A, it is evident that between 2016 and 2019, resistance to CIP and TET was consistently higher, while resistance to ERY and GEN was lower. Similarly, in Figure 13B, isolates from animal sources also showed higher resistance levels to CIP and TET compared to ERY and GEN. When comparing the two types of isolates by year, in both 2016 and 2017, human isolates showed higher resistance to TET. However, in 2017, animal isolates showed higher resistance to CIP and ERY than human isolates.

### 2.7. Resistance of Campylobacter spp. Isolates from Oceania Studies

Data on AMR *Campylobacter* spp. isolates from Oceania, focusing on Australia, are summarized in Table 6.

In Australia, 281 human and 237 animal isolates were studied. Between 2019 and 2020, resistance to CIP and TET decreased among human isolates, while resistance to ERY increased. By 2020, CIP had the highest resistance among human isolates. In 2012, TET was the antibiotic with the highest resistance among animal isolates.

**Figure 14:**
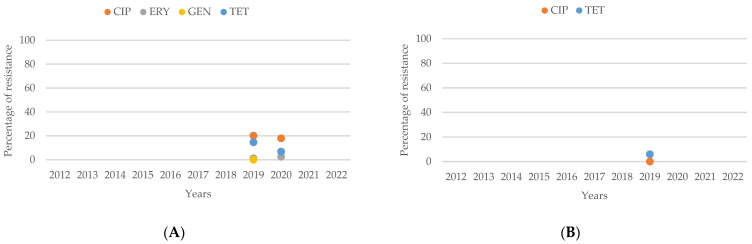
(**A**) Antibiotic resistance patterns of *Campylobacter* spp. isolates from humans from Oceania between 2012 and 2022. (**B**) Antibiotic resistance patterns of *Campylobacter* spp. isolates from animals from Oceania between 2012 and 2022.

In 2019, comparing the resistance percentages between human and animal isolates in Figure 14A,B of *Campylobacter* spp. in Australia, it was observed that the percentages of resistance to both CIP and TET were higher in human isolates compared to animal isolates. This highlights a greater prevalence of resistance to these antibiotics in human cases relative to those found in animals during that period.

## 3. Discussion

There is evidence suggesting a global increase in the incidence of campylobacteriosis [154]. The present study reviews the status of resistance of *Campylobacter* spp. worldwide to commonly used antibiotics.

### 3.1. Global Patterns and Regional Discrepancies in Campylobacter spp. Infections

The incidence of *Campylobacter* spp. infections has been increasing in regions like North America, Europe, and Australia. Epidemiological data from regions such as Africa and Asia, while incomplete, suggest that *Campylobacter* spp. infection is endemic in these areas. The number of reported cases and the frequency of resistance vary widely across different countries and even within regions of the same country [155]. These variations can be attributed to several factors, including differences in detection methodologies, geographic and population differences, the extent and focus of case studies, variations in biocontrol protocols, surveillance practices, and dietary habits, and the presence of natural reservoirs for *Campylobacter* species. The review covered a wide geographical distribution with studies from Africa, Asia, Europe, North America, South America, and Oceania, highlighting the global concern of antimicrobial resistance in *Campylobacter* spp. isolates.

It is worth mentioning that more than 50% of the studies on human isolates of *Campylobacter* spp. were conducted in the Americas. In contrast, most studies on animal isolates of *Campylobacter* spp. were carried out in Europe. This discrepancy may be due to several factors. In the USA and Canada, there is a robust system for the surveillance and research of foodborne illnesses, which includes monitoring *Campylobacter* spp. in humans. Programs like FoodNet in the USA are dedicated to identifying and studying foodborne pathogens, resulting in a higher number of human isolates. Additionally, the advanced public health infrastructure in North America facilitates the collection, analysis, and reporting of data from human isolates, contributing to a greater quantity of available studies and samples.

In Europe, there is a strong emphasis on animal food safety and the control of zoonoses, leading to the extensive monitoring of *Campylobacter* in production animals such as poultry, pigs, and cattle. This is regulated by European Union programs aimed at ensuring food safety and animal health. Strict regulations in Europe require stringent control of pathogens in animals intended for human consumption. Initiatives such as the European Zoonosis Surveillance Program (EFSA) and the EU *Campylobacter* Reference Laboratory Network promote the collection and analysis of animal isolates.

### 3.2. MIC Testing Dominates Antimicrobial Resistance Research

MIC methods were the most used across studies for assessing resistance, indicating their importance in obtaining precise measurements of antimicrobial susceptibility. MIC values are the most used for assessing antimicrobial resistance due to their precise, quantitative measurement of the minimum concentration required to inhibit bacterial growth [156]. They provide reproducible results through standardized conditions, offer insights into antibiotic effectiveness across a range of concentrations, and directly correlate with clinical breakpoints, aiding in appropriate treatment selection [157]. Additionally, MIC testing can detect low-level resistance not always visible with qualitative methods like disk diffusion, and its flexibility allows for application across various bacterial species and antibiotics, making it a preferred choice in research and diagnostics [158].

### 3.3. Predominance of Campylobacter jejuni

Across most studies, *C. jejuni* was identified as the predominant species in both human and animal isolates, highlighting its significant role in campylobacteriosis and resistance issues. Research by Hoque et al. [159], Barata et al. [160], Zbrun et al. [161], and others found that *C. jejuni* was most associated with broiler poultry and ruminants. Conversely, *C. coli* was found to be the most prevalent in pigs, aligning with the findings of di Donato et al. [90]. The prevalence of both *C. jejuni* and *C. coli* is likely underreported, as noted by Wagennar et al. [162].

### 3.4. Antimicrobial Resistance in Campylobacter spp. Isolates in Africa

In Africa, ERY resistance was most observed in human isolates, whereas TET resistance was the most frequent among animal isolates. All MDR isolates were from animals, with Benin having the highest percentage of MDR isolates (90.8%). The region’s arid and semi-arid climate, limited healthcare access, and frequent use of antibiotics like ERY, TET, and CIP in broiler farms contribute to high resistance levels [28]. The use of fluoroquinolones, linked to increased *Campylobacter* resistance, is contrasted by lower resistance in countries where such use is banned, like Australia and Nordic Europe [30]. The relatively lower resistance to GEN may be due to its infrequent use in poultry, attributed to its high cost [163].

Despite being banned for use in farm animals for human consumption in the EU since 1994, CHL is still widely used in many developing countries, where antibiotics are often available over the counter. This continued use contributes to the exacerbation of antimicrobial resistance [23]. The high frequency of antibiotic use, often without medical oversight, is facilitated by the existence of informal markets for antibiotic sales, as noted by some authors [164,165]. Another factor contributing to this high resistance is the common practice of taking incomplete doses of antibiotics [166].

### 3.5. Antimicrobial Resistance in Campylobacter spp. Isolates in Asia

In Asia, resistance patterns varied, with decreasing percentages of resistant *Campylobacter* isolates from both human and animal sources in China, and resistant animal isolates in Japan. Equally, resistance in animal isolates increased in India, South Korea, the Philippines, and Thailand. TET was the antibiotic to which the largest number of human isolates showed resistance, while animal isolates were most resistant to CIP and TET. China had the highest percentage of MDR isolates (90.4% in 2021). Asia, being the largest continent by area and population, features diverse practices, including widespread self-medication with antibiotics, as they are readily available without any prescriptions. Extensive antibiotic use in animal farming for disease prevention, treatment, and growth promotion is common, especially in the poultry, swine, and cattle sectors [58]. This likely contributes to the resistance observed in *Campylobacter* isolates, with China’s high MDR prevalence values linked to intensive antimicrobial use in agriculture [37].

It is noteworthy that the highest levels of tetracycline resistance among human *Campylobacter* isolates are observed in countries from the Asian continent. This observation might be linked to the extensive use of tetracyclines in agriculture, including aquaculture and livestock practices in these regions. According to Chang et al. [167], tetracyclines are inexpensive, easy to administer, and generally have minimal side effects. The analysis of animal isolates revealed that TET is the antibiotic to which the highest number of isolates demonstrated resistance.

### 3.6. Antimicrobial Resistance in Campylobacter spp. Isolates in Europe

European countries demonstrated varied patterns. In Europe, the percentages of resistant *Campylobacter* isolates decreased in seven countries: Belgium, Croatia, Spain, and Poland for human isolates, and Italy, Poland, Portugal, and Romania for animal isolates. CIP was the antibiotic that most frequently displayed resistance phenotypes among both human and animal isolates. MDR isolates were reported in 13 countries for animal sources, with only Spain reporting MDR in human isolates. Europe maintains strict regulations on antibiotic use in humans and farm animals, with guidelines to prevent antimicrobial resistance. According to the EFSA [15], resistance levels are categorized from ‘Rare’ (<0.1%) to ‘Extremely high’ (>70%). Data from 2021 to 2022 showed high to extremely high CIP (fluoroquinolone) resistance levels (>80%) in *Campylobacter* isolates across Europe. Specifically, resistance ranged from 33.1% to 100% in human isolates and 41.7% to 84.1% in animal isolates, aligning with EFSA reports. Since CIP and other fluoroquinolones are commonly used in both human medicine and veterinary practice. This widespread use can contribute to the development of resistance in both environments. Consequently, fluoroquinolones are no longer recommended for treating *Campylobacter* infections in humans [15]. In an EFSA report [15], ERY resistance, critical for human treatment, was reported at low levels (0.9% to 7.8%) in human isolates and low to high levels (1.0% to 35.7%) in animal isolates. In the present study, in 2022, 0% of human and 5.7% of animal isolates showed ERY resistance, lower than previously reported. In an EFSA report [15], TET resistance was high to extremely high (43.3% to 90.5%) in animal isolates and extremely high (71.2%) in human isolates; the present study found 51.7% of animal and 35.1% of human isolates were resistant to TET, differing from EFSA data which indicated higher resistance in human isolates.

There are discrepancies in ERY resistance reports, with the EFSA indicating low to moderate levels, while the present study found no ERY resistance in human isolates and a slightly higher frequency of resistance in animal isolates. For TET, both sources concur on high to extremely high resistance in animal isolates; however, the study reports significantly lower resistance in human isolates compared to EFSA data, suggesting possible regional or methodological differences in assessment.

In Europe, southern countries such as Portugal, Spain, Italy, and Greece exhibit a higher frequency of resistance to antibiotics like CHL and ERY in *Campylobacter* isolates compared to northern countries such as Estonia and Latvia. Resistance to CIP and TET is widespread, while GEN shows the lowest frequency of resistance, with the highest found in Latvia. Additionally, Southern Europe reports the highest percentage of MDR isolates. These regional differences suggest that specific antibiotic usage practices and environmental factors play significant roles in the observed variations in the frequency of resistance [168].

### 3.7. Antimicrobial Resistance in Campylobacter spp. Isolates in North and Central America

Canada and the USA exhibit similar patterns, with an increasing frequency of resistance observed in animal isolates and, in Canada, also in human isolates. TET and CIP are the antibiotics to which the highest number of human isolates showed resistance, while TET was the most common antibiotic for resistance in animal isolates. Only animal isolates were classified as MDR, with the USA reporting the highest percentage of MDR isolates at 16.7% in 2022.

The Canadian Integrated Program for Antimicrobial Resistance Surveillance (CIPARS) has consistently identified *C. jejuni* and *C. coli* as the predominant *Campylobacter* species in human isolates, with *C. jejuni* representing 91% and *C. coli* representing 7% of tested isolates [169]. The program has historically noted high levels of resistance to CIP and TET among *Campylobacter* spp. isolates. Although GEN resistance was not previously observed in animals or food components, it has been detected since 2019, marking a significant shift in resistance patterns. This recent emergence of GEN resistance aligns with the present study’s findings, which indicate that CIP and TET are the antibiotics to which the highest percentages of human isolates exhibit resistance. Additionally, previous reports, such as those by Webb and colleagues [130], have documented resistance issues in animal isolates from beef cattle, suggesting that the problem of GEN resistance may have been underreported earlier. These patterns highlight the ongoing need for vigilant surveillance and may necessitate a reevaluation of current treatment approaches to effectively manage emerging AMR.

The higher percentage of resistant *Campylobacter* isolates in Guatemala can be directly related to the widespread availability of antibiotics without a prescription. Antibiotic use in Guatemala is frequently marked by inappropriate practices, such as over-prescription and indiscriminate use, with antibiotics often being purchased without a prescription [165]. This practice contributes to the excessive use of antibiotics and, consequently, to the selection and proliferation of resistant strains [166]. In contrast, more stringent control over the prescription and sale of antibiotics in the USA and Canada helps to minimize the selective pressure that contributes to antimicrobial resistance.

### 3.8. Antimicrobial Resistance in Campylobacter spp. Isolates in South America

In South America, appropriate antibiotic use faces serious challenges due to inadequate practices and regulatory deficiencies. Among the four countries studied, three reported increased percentages of resistant isolates from both human and animal sources. For human isolates, resistance was highest for CIP and TET, whereas in animal isolates, CIP was the most common resistance observed.

Excessive and indiscriminate use of antibiotics is common, with self-medication and purchasing medications without a prescription contributing to the rise in antimicrobial resistance [144]. The lack of strict regulation and the uncontrolled sale of antibiotics in pharmacies exacerbates this problem. Resistance to antibiotics, such as CIP, TET, and ERY, is increasing, reflecting improper use of these medications in healthcare and livestock settings. Inadequate public health infrastructure and medical education also contribute to inappropriate prescribing practices [150]. Despite efforts in some countries to improve regulation and promote rational antibiotic use, effective implementation of policies and practices is often limited by resource constraints and the need for cultural and educational changes [146].

### 3.9. Regional MDR Variations

MDR is prevalent in animal isolates. However, there is a significant regional variation in MDR prevalence. A higher MDR prevalence is described in Portugal, a country in the south of Europe, and a lower MDR prevalence corresponds to a study from the northern European country Finland. In China, the prevalence is also high, while in Thailand it is low. The variations observed underscore the necessity for tailored, region-specific strategies to address resistance effectively.

### 3.10. Global Patterns in Antimicrobial Resistance

In many regions, there has been a notable increase in interest in resistance to critical antibiotics, due to their significant roles in treating infections and the implications of resistance, such as for CIP and TET. For example, in North America, both Canada and the US have observed rising resistance in *Campylobacter* isolates, with TET and CIP being the most frequently observed resistance phenotypes. This trend indicates a growing challenge in managing infections and highlights the need for ongoing surveillance and more stringent antimicrobial stewardship practices.

Temporal patterns can vary significantly across regions. In Europe, for instance, the frequency of resistance to CIP has been consistently high, while in other regions like South America and Asia, resistance patterns have shown more variability. These differences underscore the impact of regional practices, such as antibiotic use in agriculture and human medicine, on resistance prevalence.

In all the mentioned studies, it was evident that the antibiotics CIP and TET displayed the highest levels of antimicrobial resistance among isolates of *Campylobacter*, whether they were of human or animal origin. However, depending on the origin and type of isolate, the highest percentage varies. In general, considering the antibiotic with the highest percentage of resistance in the most recent study from each country, TET emerged as the most prevalent, being reported in 26 studies, followed closely by CIP, which was reported in 25 studies. ERY was reported by 13 countries, while CHL was reported by only one. GEN was never reported as the antibiotic with the highest percentage of resistance in the latest study from any country. For human isolates, the highest frequency of resistance was to CIP in European and North American countries. This was to be expected since FDA approved CIP for, among others, the treatment of gastrointestinal infections [170,171,172]. The same type of treatment is practiced in Canada [173]. According to Adriaenssens et al. [174], the same happens in Europe, since CIP is the most consumed second-generation quinolone in 24 European countries.

Resistance levels generally increased across most continents, with Africa, Asia, Europe, and North and Central America showing more increases than decreases. However, South America and Oceania had more decreases than increases, with South America seeing 11 decreases compared to 2 increases and Oceania seeing 1 decrease versus 2 increases.

## 4. Materials and Methods

### 4.1. Literature Study Search and Selection Strategy

A comprehensive systematic literature search in the b-on (Portuguese Online Knowledge Library), ScienceDirect, PubMed, and Google Scholar databases was performed to access studies published between 2012 and 2022 using the following search keywords: *Campylobacter* spp. AND (antimicrobial resistance OR antibiotic resistance OR resistant isolates). From then on, all studies in the search were analyzed by title, abstract, and full text. Full-text articles and published reviews were collected, and conference abstracts and book chapters were excluded. Only studies in English or Spanish were considered for full review if: (1) it was possible to obtain the full article, (2) the article reported one of two techniques: disk diffusion (DD) or minimum inhibitory concentration (MIC) in *Campylobacter* spp., and (3) it reported studies of *Campylobacter* spp. from isolates of human, broiler, pig, and/or ruminant origin.

Once the eligibility criteria were met, the selected articles underwent a full-text review. Some of these articles were excluded for the following reasons: (1) they were not within the scope of this analysis, (2) they failed to provide the prevalence of resistant *Campylobacter* isolates for each type of sample analyzed, or (3) they contained unclear data.

### 4.2. Data Extraction

After a careful analysis, the following data were extracted from the text, tables, or figures of the selected publication: the surname of the first author, the year of publication, the continent, country, origin and total number of samples analyzed, the type of sampling, the species identified, the method of assessment of antimicrobial resistance, the antibiotic concentration, the number and/or percentage of isolates considered resistant, and the number of MDR isolates.

### 4.3. Antimicrobial Susceptibility Testing (AST)

The antimicrobial phenotypic resistance of *Campylobacter* spp. was evaluated using one of two AST methods: minimum inhibitory concentration (MIC) or disk diffusion (DD).

From the selected articles, particulary those that used the MIC methodology and in which the results of all isolates under study were presented, it was possible to use harmonized ECOFFs to interpret the results and make their comparison more precise, as shown in Table 7. In cases where this was not possible, the results are presented according to the criteria described by each author.

MDR refers to the resistance of a microorganism to three or more classes of antimicrobial agents commonly used to treat infections. In this study, we classified the isolates as MDR based on the definitions provided by the references cited.

### 4.4. Data Analysis

Figures containing maps were built using Microsoft^®^ Excel for Mac Version 16.88 (24081116).

## 5. Conclusions

The antimicrobial resistance of *Campylobacter* spp. continues to challenge food safety and public health. Researchers have identified several mechanisms underlying these resistances, and the findings, combined with insights into isolate associations, provide a deeper understanding of the development and dissemination of antibiotic resistance, as well as the transmission of these microorganisms to new hosts.

Globally, the frequency of resistance is gradually increasing across multiple antibiotics, leading to a pattern of multiple resistance. The high prevalence of fluoroquinolone (CIP) resistance has significantly reduced the effectiveness of these drugs in treating campylobacteriosis in humans. Given the relation between human, animal, and environmental health under the One Health concept, investigating the factors influencing resistance transmission and maintenance in *Campylobacter* spp. across various environments and hosts is imperative.

Moreover, in the context of world trade, the movement of animals and food products across borders can facilitate the global spread of antibiotic resistance. Understanding and addressing this challenge is crucial to mitigating the risks associated with AMR in international markets.

Despite most studies not determining MDR in human isolates, a human isolate in 2021 was considered MDR, and the future progression of AMR in *Campylobacter* spp. isolates remains a significant concern. Improved surveillance systems and data-sharing networks will be crucial for the early detection and monitoring of emerging resistance patterns. Furthermore, the development of novel antimicrobial agents and alternative treatment strategies could offer new solutions to combat resistance. Promoting global stewardship programs and integrating AMR considerations into agricultural and healthcare practices will also be essential. By adopting a collaborative One Health approach and investing in research and innovation, we can work towards mitigating the impact of AMR and protecting public health for future generations.

## Figures and Tables

**Table 1:** Results concerning *Campylobacter* spp. isolates resistance to antimicrobials from African studies.

Country	No. of Isolates	Aim of Sampling	First Study	Last Study	AMR Increase	AMR Decrease	Higher in Last Study	% of MDR	Source
Benin	109 from animals	Focused sampling	2022	2022	Na	Na	TET	90.8	[16]
Ethiopia	48 from humans	Passive surveillance of diagnostic samples Structured survey	2014	2021	CIP ERY GEN TET	CHL	TET	Nd	[17,18]
	91 from animals	Structured survey Focused sampling Monitoring sample	2021	2022	CHL	CIP TET	CHL	84.2	[18,19,20]
Ivory Coast	76 from animals	Focused sampling	2012	2012	Na	Na	CIP	Nd	[21]
Kenya	18 from humans	Passive surveillance of diagnostic samples	2021	2021	Na	Na	ERY	Nd	[22]
	35 from animals	Focused sampling	2016	2016	Na	Na	CIP	54.3	[23]
Morocco	143 from animals	Focused sampling	2020	2020	Na	Na	TET	Nd	[24]
South Africa	464 from animals	Monitoring samples	2020	2021	CHL CIP ERY GEN TET	None	ERY	87.3	[25,26]
Tanzania	136 from humans	Passive surveillance of diagnostic samples	2015	2015	Na	Na	ERY	Nd	[27]
	134 from animals	Focused sampling Survey sampling	2015	2016	CIP ERY	GEN	ERY	47.6 (in 2016)	[28,29]
Tunisia	180 from animals	Focused sampling Monitoring samples	2018	2022	Na	CIP ERY	TET	37.5 (in 2022)	[30,31]

First study—the year of the first study selected for this research. Last study—the year of the last study selected for this research. AMR increase—antibiotics for which the percentage of resistant isolates increased between the first and last year selected for this research. AMR decrease—antibiotics for which the percentage of resistant isolates decreased between the first and last year selected for this research. Higher in last study—antibiotics for which the percentage of resistant isolates was higher in the last year of the study compared to any other year selected for this research. Na—Not applicable. Nd—Not determined.

**Table 2:** The results of the investigation of resistant *Campylobacter* spp. isolates in Asia.

Country	No. of Isolates	Aim of Sampling	First Study	Last Study	AMR Increase	AMR Decrease	Higher in Last Study	% of MDR	Source
China	805 from humans	Passive surveillance of diagnostic samples Monitoring samples Focused sampling	2014	2022	TET	CHL CIP ERY GEN	TET	Nd	[32,33,34,35,36]
	755 from animals	Focused sampling Structured surveys Monitoring samples	2014	2022	ERY GEN	CHL CIP TET	CIP	90.4 (in 2021)	[32,37,38,39,40]
India	36 from humans	Passive surveillance of diagnostic samples	2013	2013	Na	Na	CIP	Nd	[41]
	508 from animals	Focused sampling	2018	2021	CIP ERY GEN TET	Na	TET	41.5 (in 2021)	[42,43]
Japan	430 from humans	Passive surveillance of diagnostic samples	2019	2019	Na	Na	CIP	Nd	[44]
	602 from animals	Structured surveys Focused sampling	2012	2017	Na	CHL ERY	TET	Nd	[45,46,47,48]
Jordan	38 from humans	Monitoring samples	2012	2012	Na	Na	CIP ERY TET	Nd	[49]
Korea	121 from humans	Passive surveillance of diagnostic samples	2013	2013	Na	None	ERY	87.3	[50]
	1721 from animals	Focused sampling Monitoring samples Structured surveys	2017	2021	CHL CIP ERY GEN TET	None	CIP	75.5 (in 2021)	[51,52,53,54,55,56]
Pakistan	80 from humans	Focused sampling	2018	2018	Na	Na	TET	Nd	[57]
Philippines	251 from animals	Focused sampling Monitoring samples	2014	2017	ERY GEN TET	Na	TET	71.4 (in 2014)	[58,59]
Thailand	215 from animals	Focused sampling Structured surveys	2013	2021	CIP ERY GEN TET	Na	CIP	15.3 (in 2021)	[60,61]

First study—the year of the first study selected for this research. Last study—the year of the last study selected for this research. AMR increase—antibiotics for which the percentage of resistant isolates increased between the first and last year selected for this research. AMR decrease—antibiotics for which the percentage of resistant isolates decreased between the first and last year selected for this research. Higher in last study—antibiotics for which the percentage of resistant isolates was higher in the last year of the study compared to any other year selected for this research. Na—Not applicable. Nd—Not determined.

**Table 3:** The results of the investigation of resistant *Campylobacter* spp. isolates in Europe.

Country	No. of Isolates	Aim of Sampling	First Study	Last Study	AMR Increase	AMR Decrease	Higher in Last Study	% of MDR	Source
Austria	55 from animals	Focused sampling	2016	2016	Na	Na	CIP	12.7	[62]
Belgium	472 from humans	Passive surveillance of diagnostic samples	2017	2020	Na	CIP ERY TET	CIP	Nd	[63,64,65]
	249 from animals	Focused sampling	2018	2019	CIP ERY TET	GEN	TET	5.4 (in 2018)	[64,66]
Croatia	65 from humans	Passive surveillance of diagnostic samples	2020	2022	Na	CIP TET	CIP	Nd	[67,68]
	51 from animals	Focused sampling	2020	2020	Na	Na	CIP	Nd	[67]
Czechia	23 from humans	Passive surveillance of diagnostic samples	2018	2018	Na	Na	CIP	Nd	[69]
	103 from animals	Focused sampling	2018	2018	Na	Na	CIP	60.0	
Estonia	15 from humans	Passive surveillance of diagnostic samples	2022	2022	Na	Na	CIP	Nd	[70]
	4 from animals	Focused sampling	2022	2022	Na	Na	Na	Nd	
Finland	95 from humans	Focused sampling	2016	2016	Na	Na	CIP	Nd	[71]
	579 from animals	Monitoring samples	2016	2016	Na	Na	CIP	0.2	
France	2416 from humans	Monitoring samples	2015	2015	Na	Na	CIP	Nd	[72]
	276 from animals	Focused sampling Structured surveys	2015	2017	ERY GEN TET	CIP	TET	61.5 (2015)	[73,74]
Germany	737 from animals	Focused sampling Monitoring samples	2012	2021	CIP ERY GEN TET	Na	CIP	Nd	[75,76,77]
Greece	276 from animals	Focused sampling Monitoring samples Structured surveys	2015	2017	ERY GEN TET	CIP	TET	61.5 (in 2015)	[78,79,80,81,82,83,84]
Ireland	96 from animals	Monitoring samples	2022	2022	Na	Na	TET	15.6	[85]
Italy	51 from humans	Structured surveys	2019	2019	Na	Na	CIP	Nd	[86]
	1197 from animals	Structured surveys Focused sampling Monitoring samples	2014	2021	None	CHL CIP ERY GEN TET	TET	61.8	[87,88,89,90,91,92]
Latvia	51 from animals	Structured surveys Focused sampling	2014	2022	ERY GEN TET	Na	CIP	12.5	[70,93]
Lithuania	26 from animals	Focused sampling	2022	2022	Na	Na	CIP	38.5	[70]
Poland	297 from humans	Passive surveillance of diagnostic samples Structured surveys Focused sampling	2017	2020	CIP ERY GEN TET	Na	CIP	Nd	[94,95,96]
	3689 from animals	Structured surveys Focused sampling Monitoring samples	2013	2022	GEN	CIP ERY TET	TET	8.1 (in 2022)	[94,95,97,98,99,100,101,102,103,104]
Portugal	763 from animals	Structured surveys Focused sampling	2012	2022	CIP TET	CHL ERY GEN	CIP	98.5 (in 2022)	[105,106,107,108,109]
Romania	111 from animals	Monitoring samples Passive surveillance of diagnostic samples	2020	2022	TET	CIP ERY	CIP	6.9 (in 2022)	[110,111]
Spain	139 from humans	Focused sampling	2014	2021	Na	CIP ERY GEN TEY	ERY	3.8 (in 2021)	[112,113,114,115]
	5402 from animals	Monitoring samples Focused sampling Structured surveys	2013	2021	CIP ERY GEN TET	Na	TET	12.5 (in 2021)	[113,114,115,116,117,118,119,120,121,122,123]
Sweden	215 from animals	Focused sampling	2017	2021	TET	CIP	CIP	Nd	[74,124]
UK	41 from animals	Focused sampling	2014	2014	Na	Na	TET	Nd	[125]

First study—the year of the first study selected for this research. Last study—the year of the last study selected for this research. AMR increase—antibiotics for which the percentage of resistant isolates increased between the first and last year selected for this research. AMR decrease—antibiotics for which the percentage of resistant isolates decreased between the first and last year selected for this research. Higher in last study—antibiotics for which the percentage of resistant isolates was higher in the last year of the study compared to any other year selected for this research. Na—Not applicable. Nd—Not determined.

**Table 4:** Results of the investigation of resistant *Campylobacter* spp. isolates in North and Central America.

Country	No. of Isolates	Aim of Sampling	First Study	Last Study	AMR Increase	AMR Decrease	Higher in Last Study	% of MDR	Source
Canada	749 from humans	Passive surveillance of diagnostic samples	2013	2015	CIP ERY GEN TET	Na	TET	Nd	[126,127,128]
	1951 from animals	Structured surveys Monitoring samples Passive surveillance of diagnostic samples	2012	2019	CIP ERY GEN	TET	TET	4.3 (in 2012)	[129,130,131]
Grenada	162 from animals	Structured surveys	2014	2014	Na	Na	Na	Nd	[132]
Guatemala	161 from humans	Passive surveillance of diagnostic samples	2013	2013	Na	Na	TET	Nd	[133]
Mexico	360 from humans	Structured surveys	2012	2012	Na	Na	CIP	Nd	[134]
	2698 from animals	Focused sampling	2012	2012	Na	Na	TET	Nd	
USA	11,726 from humans	Passive surveillance of diagnostic samples	2017	2017	Na	Na	CIP	Nd	[135]
	1396 from animals	Focused sampling Structured survey Passive surveillance of diagnostic samples	2014	2022	CIP ERY GEN TET	Na	TET	16.7 (in 2022)	[125,135,136,137,138,139,140]

First study—the year of the first study selected for this research. Last study—the year of the last study selected for this research. AMR increase—antibiotics for which the percentage of resistant isolates increased between the first and last year selected for this research. AMR decrease—antibiotics for which the percentage of resistant isolates decreased between the first and last year selected for this research. Higher in last study—antibiotics for which the percentage of resistant isolates was higher in the last year of the study compared to any other year selected for this research. Na—Not applicable. Nd—Not determined.

**Table 5:** The results of the investigation of resistant *Campylobacter* spp. isolates in South America.

Country	No. of Isolates	Aim of Sampling	First Study	Last Study	AMR Increase	AMR Decrease	Higher in Last Study	% of MDR	Source
Brazil	176 from animals	Focused sampling Monitoring samples	2013	2020	None	CHL CIP ERY GEN TET	CIP	35.7 (in 2020)	[141,142,143,144,145]
Chile	7 from humans	Structured surveys	2016	2016	Na	Na	CIP TET	Nd	[146]
	347 from animals	Focused sampling	2016	2017	ERY	CIP GEN TET	ERY	Nd	[146,147]
Ecuador	218 from animals	Structured surveys	2017	2017	Na	Na	CIP	Nd	[148]
Peru	1032 from animals	Focused sampling Structured surveys	2017	2019	GEN	CIP ERY TET	CIP	Nd	[149,150]

First study—the year of the first study selected for this research. Last study—the year of the last study selected for this research. AMR increase—antibiotics for which the percentage of resistant isolates increased between the first and last year selected for this research. AMR decrease—antibiotics for which the percentage of resistant isolates decreased between the first and last year selected for this research. Higher in last study—antibiotics for which the percentage of resistant isolates was higher in the last year of the study compared to any other year selected for this research. Na—Not applicable. Nd—Not determined.

**Table 6:** The results of the investigation of resistant *Campylobacter* spp. isolates in Oceania.

Country	No. of Isolates	Type of Sampling	First Study	Last Study	AMR Increase	AMR Decrease	Higher in Last Study	% of MDR	Source
Australia	281 from humans	Passive surveillance of diagnostic samples Structured surveys	2019	2020	ERY	CIP TET	CIP	Nd	[151,152]
	237 from animals	Monitoring samples	2012	2012	Na	Na	TET	Nd	[153]

First study—the year of the first study selected for this research. Last study—the year of the last study selected for this research. AMR increase—antibiotics for which the percentage of resistant isolates increased between the first and last year selected for this research. AMR decrease—antibiotics for which the percentage of resistant isolates decreased between the first and last year selected for this research. Higher in last study—antibiotics for which the percentage of resistant isolates was higher in the last year of the study compared to any other year selected for this research. Na—Not applicable. Nd—Not determined.

**Table 7:** Panel of antimicrobial substances included in AMR monitoring and thresholds for interpreting resistance of *C. jejuni* and *C. coli* based on Commission Implementing Decision (EU) 2020/1729 and EFSA Technical Report 2021.

Antimicrobial	*C. jejuni* EU Surveillance 2021 EUCAST ECOFF	*C. coli* EU Surveillance 2021 EUCAST ECOFF
Chloramphenicol (CHL)	>16	>16
Ciprofloxacin (CIP)	>0.5	>0.5
Erythromycin (ERY)	>4	>8
Gentamicin (GEN)	>2	>2
Tetracycline (TET)	>1	>2

## Data Availability

Not applicable.
